# Delayed Dynamic Abdominal Wall Closure Following Congenital Diaphragmatic Hernia Repair Using a Gore-Tex^®^ Patch: A Single-Center Retrospective Experience for a Complex Pediatric Thoracic Disease

**DOI:** 10.3390/children13091133

**Published:** 2026-08-24

**Authors:** Lucas Moratilla-Lapeña, José Luis Encinas, Eugenia Antolín, Ana Sánchez Torres, Francisco Hernández

**Affiliations:** 1 Pediatric Surgery Department, Hospital Universitario La Paz, 28046 Madrid, Spain; lucas.moratilla@salud.madrid.org (L.M.-L.);; 2 Fetal Medicine Unit, Obstetrics and Gynecology Department, Hospital Universitario La Paz, 28046 Madrid, Spain; 3 Neonatology Department, Hospital Universitario La Paz, 28046 Madrid, Spain; 4 La Paz Research Institute (Idipaz), Hospital Universitario La Paz, 28046 Madrid, Spain; 5 European Reference Network on Transplantation in Children (TransplantChild ERN), 28046 Madrid, Spain

**Keywords:** congenital diaphragmatic hernia, delayed abdominal closure, intra-abdominal pressure, abdominal compartment syndrome

## Abstract

**Highlights:**

**What are the main findings?**

- Delayed dynamic abdominal wall closure using a Gore-Tex^®^ patch with dynamic traction under intra-abdominal pressure monitoring achieved early definitive fascial closure (median 6 days) in CDH neonates unsuitable for primary closure.
- Patients requiring delayed dynamic abdominal wall closure presented significantly more severe baseline disease (lower O/E LHR, more liver herniation, more advanced defect type, greater ECMO need) than those undergoing primary closure.

**What are the implications of the main findings?**

- The higher mortality observed in the delayed dynamic abdominal wall closure group was strongly associated with markers of baseline disease severity; however, given the small sample size and the lack of adjusted multivariable analysis, the independent contribution of the closure technique itself cannot be excluded.
- These findings align with prior reports linking abdominal wall patch requirement to greater baseline severity and mortality risk, supporting further evaluation of dynamic traction closure as a feasible option for definitive fascial closure in this high risk subgroup.

**Abstract:**

Introduction: delayed abdominal closure following congenital diaphragmatic hernia (CDH) repair is required in patients with large defects in whom primary fascial closure would risk abdominal compartment syndrome. Several strategies have been described, but most require prolonged time to definitive closure. We present a novel technique based on a Gore-Tex^®^ abdominal patch with dynamic traction under intra-abdominal pressure monitoring. Methods: retrospective observational study of 41 neonates undergoing CDH repair at a tertiary referral center (2015–2023). Patients were divided into primary closure (*n* = 27) and delayed dynamic abdominal wall closure (DDAC) (*n* = 14) groups. Baseline characteristics, surgical outcomes, and mortality were compared between groups using Fisher’s exact test and the Wilcoxon rank-sum test. Results: patients requiring DDAC had more severe baseline disease, including lower O/E LHR (26.30 [21.40–31.00] vs. 42.00 [34.00–46.00], *p* < 0.001), higher rates of liver herniation (13/14 vs. 4/27, *p* < 0.001), more advanced defect type (*p* < 0.001) and greater need of ECMO (9/14 vs. 3/27, *p* < 0.001). Definitive fascial closure was achieved at a median of 6 days. In-hospital mortality after diaphragmatic repair was 19.5% (8/41), with 7 deaths occurring in the DDAC group. Mortality was strongly associated with the gradient of disease severity across prenatal and anatomical markers, although an independent contribution of the abdominal closure strategy cannot be excluded. Conclusions: DDAC achieves early definitive fascial closure and appears technically feasible in CDH patients unsuitable for primary closure. Higher mortality in this group was strongly associated with baseline disease severity, though its independent relationship with the closure technique remains to be established.

## 1. Introduction

Congenital diaphragmatic hernia (CDH) is a rare congenital anomaly characterized by incomplete diaphragm fusion, resulting in herniation of abdominal viscera into the thoracic cavity. This leads to impaired pulmonary development and pulmonary circulatory dysfunction, which may prove fatal despite surgical correction. Extensive research has focused on its pathogenesis, prognostic factors, and intra- and extrauterine therapies, as well as defining the optimal timing for surgical repair to improve survival [1].

Surgical correction of this condition involves closing the diaphragmatic defect, which can be done either directly or by using various types of patches [2]. Sometimes, the reduction of the abdominal viscera into the abdominal cavity is impossible due to a lack of space within it, caused by their migration into the thoracic cavity during the fetal period. As a result, primary closure of the abdominal wall is unlikely during a first-stage procedure without causing abdominal compartment syndrome (ACS). ACS is a potentially fatal condition, with reported mortality rates of up to 60%, caused by sustained intra-abdominal pressure (IAP) elevation above 10 mmHg, leading to visceral and lower limb hypoperfusion, respiratory failure, hemodynamic instability, and renal dysfunction [3,4]. Several strategies have been described to address this challenge, including biological and synthetic patches, silo construction and simple skin closure. However, complete fascial closure is achieved after a prolonged period, with a mean time of approximately three months following the initial repair [5,6,7,8,9,10].

At our institution, we have developed a novel approach consisting of a Gore-Tex^®^ patch with sequential traction under continuous intra-abdominal pressure monitoring allowing complete fascial closure within the first postoperative days while avoiding the complications associated with prolonged delayed closure. The aim of this study was to describe our institutional experience with dynamic delayed abdominal wall closure (DDAC) in a CDH cohort and to compare clinical characteristics and outcomes between patients requiring DDAC and those undergoing primary abdominal closure.

## 2. Methods

### 2.1. Study Design and Patient Population

We conducted a retrospective observational study of all neonates undergoing surgical repair of CDH at a tertiary referral center for CDH (Hospital Universitario La Paz, Madrid, Spain) between January 2015 and December 2023.

Inclusion criteria were: (1) surgical repair of CDH during the neonatal period (age < 1 month) and (2) admission to the Neonatal Intensive Care Unit (NICU). Patients were excluded if they lacked prenatal diagnosis, underwent primary thoracoscopic repair, underwent delayed surgical repair (defined as repair performed after NICU discharge) or died prior to surgical correction.

Delayed abdominal wall closure was defined as any abdominal wall closure not achieved during the same surgical procedure as diaphragmatic repair. Patients not requiring delayed closure underwent primary fascial closure at the time of diaphragmatic hernia repair.

### 2.2. Data Collection and Study Variables

Data were collected through a retrospective review of electronic medical records. The study was approved by the Institutional Ethics Committee of Hospital Universitario La Paz (PI-6678 2025.317). The following variables were recorded: demographic data (sex, gestational age, birth weight); prenatal severity markers (O/E LHR, O/E LHR category); fetal therapy (FETO); neonatal condition at birth (Apgar score at 1 and 5 min); respiratory and hemodynamic support (need for high-frequency oscillatory ventilation [HFOV], need for ECMO and duration, day of ECMO initiation and if CDH repair was done while on ECMO); surgical variables (time to surgery, hernia laterality, defect type, diaphragmatic closure type, herniated content [defined as liver-up or not liver-up], abdominal closure strategy [primary closure vs. DDAC] and time to abdominal wall closure); postoperative outcomes (duration of invasive mechanical ventilation, time to initiation of enteral feeding, time to full enteral feeding, length of stay, oxygen dependency at discharge, chest wall malformation, scoliosis, and follow-up time). Defect type was classified according to the Congenital Diaphragmatic Hernia Study Group (CDHSG) staging system (A–D) [11]. O/E LHR severity was categorized as extreme (<15%), severe (15–25%), moderate (26–35%, or 36–45% in the presence of liver herniation), or mild (36–45%, or ≥46% in the presence of liver herniation), according to Deprest et al. [12]. Scoliosis was diagnosed based on a Cobb angle > 10° on follow-up imaging [13], and chest wall malformation was diagnosed clinically in the presence of pectus excavatum or carinatum. Imaging was not performed systematically at predefined intervals in either group but was obtained when clinically indicated, based on physical examination findings or incidental findings on routine chest radiographs performed for CDH follow-up. Mortality was defined as death occurring during the index hospitalization after diaphragmatic defect repair.

### 2.3. Surgical Technique

All patients underwent a left subcostal laparotomy approach. Diaphragmatic defect closure was performed primarily using interrupted Prolene 2/0 sutures in eligible patients, or with a Gore-Tex^®^ patch fixed with interrupted Prolene 2/0 sutures in those with large defects precluding primary repair.

**Figure 1:**
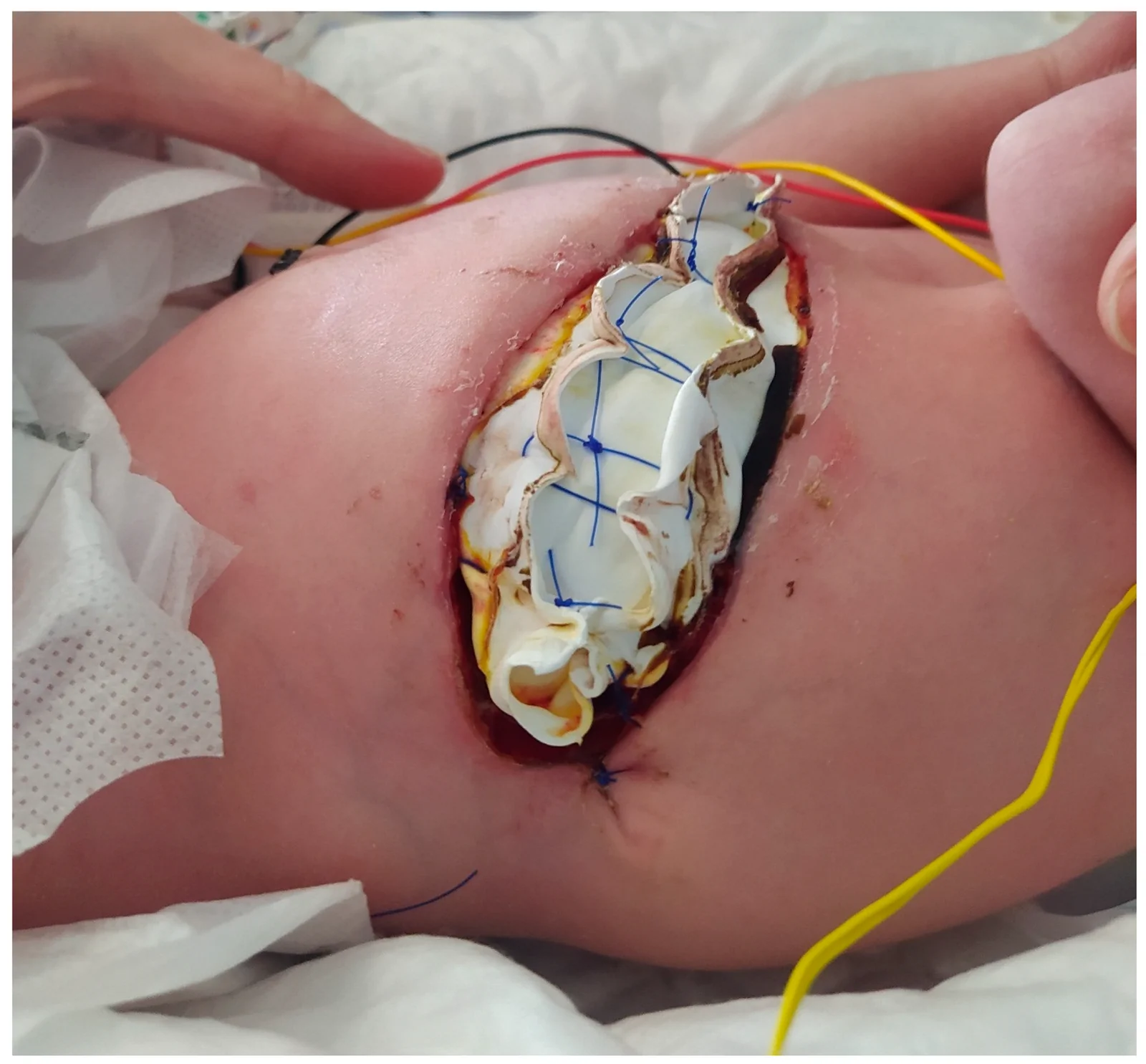
Gore-Tex^®^ abdominal patch sutured to the fascial edges, showing the redundant flap used for progressive traction and dynamic delayed abdominal wall closure.

Primary fascial approximation was attempted in all cases after diaphragmatic repair. Delayed abdominal closure was performed either when primary approximation was not feasible or when IAP, measured via a bladder catheter after closure, exceeded the safety threshold of 10 mmHg. In both scenarios, closure consisted of suturing a Gore-Tex^®^ patch (W. L. Gore & Associates, Inc.; Newark, DE, USA) to the fascial edges with a running Prolene 2/0 suture (Ethicon, Inc. (Johnson & Johnson); Raritan, NJ, USA) leaving a redundant flap at the edge to allow subsequent dynamic traction sutures (Figure 1).

Traction was started on postoperative day 1 or later, at the discretion of the surgical team based on the patient’s clinical condition. Traction was applied by placing sutures along the edge of the redundant patch flap, progressively tensioning the knots while avoiding excessive tension across the patch. Following each traction maneuver, IAP was measured via a transvesical technique through a bladder catheter, with the patient in the supine position and under sedation, without neuromuscular blockade. The bladder was first emptied and the pressure transducer was zeroed at the level of the pubic symphysis, open to atmospheric pressure. The bladder was then instilled with saline at 1 mL/kg, as previously described by Defontaine et al. [14] and IAP was subsequently measured.

A maximum threshold of 12 mmHg, rather than the 10 mmHg threshold used to indicate delayed closure, was accepted during approximation, as this represented a transient elevation in intra-abdominal pressure. Under routine conditions, IAP was measured every 2 h, but if IAP after approximation exceeded 10 mmHg, monitoring frequency was increased to every 10 min during the first hour. If pressure did not decrease to 10 mmHg or below within this period, the approximation sutures were released and a new approximation was attempted 24 h later. During this period, patients were closely monitored for reduced urine output or other signs of end-organ dysfunction. Sequential traction was repeated every 24 h until complete approximation of the fascial edges was achieved, at which point the abdominal patch was removed and definitive fascial closure was performed using Vicryl 2/0 sutures (Ethicon, Inc. (Johnson & Johnson); Raritan, NJ, USA).

This technique was already established at our institution prior to the study period and all patients requiring delayed abdominal closure during the study were managed using it.

### 2.4. Statistical Analysis

Statistical analysis was performed using RStudio 2024.12.0 (Posit Software, PBC; Boston, MA, USA) within the R environment (version 4.5.3). The normality of data distribution was assessed using the Shapiro–Wilk test. Continuous variables are presented as median and interquartile range (IQR), while categorical variables are expressed as absolute frequencies and percentages. Comparisons between groups were performed using Fisher’s exact test for categorical variables and the Wilcoxon rank-sum test for continuous variables. A *p*-value < 0.05 was considered statistically significant.

### 2.5. Declaration of Generative AI and AI-Assisted Technologies in the Manuscript Preparation Process

During the preparation of this work, the author(s) used Claude (Anthropic, PBC; San Francisco, CA, USA) in order to check and correct the grammar and language of the manuscript. After using this tool/service, the author(s) reviewed and edited the content as needed and take(s) full responsibility for the content of the published article.

## 3. Results

### 3.1. Patient Population

During the study period, 52 patients with CDH were initially assessed, of whom 41 met the inclusion criteria and were included in the final analysis. Eleven patients were excluded: three died before surgical repair (one while on ECMO and two who were not ECMO candidates), five had a postnatal diagnosis of CDH without a prenatal diagnosis and three underwent thoracoscopic repair.

The final cohort comprised 27 patients (65.9%) who underwent primary abdominal closure and 14 (34.1%) who required delayed abdominal closure. Patient distribution by year of inclusion and closure strategy did not differ significantly over the study period (*p* = 0.2; Table S1). Baseline characteristics are summarized in Table 1.

Patients requiring delayed closure presented significantly more severe disease at baseline, with lower gestational age (*p* = 0.008), lower O/E LHR (26.30 [21.40–31.00] vs. 42.00 [34.00–46.00], *p* < 0.001), higher rates of FETO (42.9% vs. 3.7%, *p* = 0.004), liver herniation (92.9% vs. 14.8%, *p* < 0.001), more advanced defect type (*p* < 0.001) (Table 2), greater need for high-frequency oscillatory ventilation (HFOV) (78.6% vs. 37.0%, *p* = 0.020) and ECMO (64.3% vs. 11.1%, *p* < 0.001). Overall, 1 of the 3 ECMO patients in the primary closure group underwent CDH repair while on ECMO, compared to 4 of the 9 ECMO patients in the DDAC group.

The median time to discharge among survivors was 31 (17–68) days, with no significant difference between the DDAC and primary closure groups (42.5 [21.0–87.0] vs. 30 [17.0–42.0] days; *p* = 0.3). Similarly, the follow-up duration did not differ significantly between groups (85 [55–122] vs. 101 [68–119] months; *p* = 0.8).

### 3.2. Surgical Outcomes

In-hospital mortality following surgical repair was 19.5% (8/41). Seven deaths (87.5%) occurred in the DDAC group, compared to one (12.5%) in the primary closure group (*p* = 0.001). Postoperative outcomes are summarized in Table 3. DDAC patients required significantly longer invasive mechanical ventilation *(p* = 0.003) and had longer oxygen dependency (*p* < 0.001). Time to first enteral feed (*p* = 0.3) and length of stay (*p* = 0.3) did not differ significantly between groups. Among survivors, time to complete enteral feeding was higher in the DDAC group compared to the primary closure group (*p* < 0.001), but the rate of home oxygen requirement at discharge was not significantly different (*p* = 0.093).

Definitive abdominal wall closure was achieved at a median of 6.00 (4.00–7.00) days from the index surgery in the DDAC group. Among patients requiring ECMO support at the time of abdominal closure, definitive fascial closure was achieved at a median of 6.5 (6.00–7.75) days, with no bleeding complications related to the procedure. These estimates are based on the 9 patients who achieved definitive closure, as the 5 patients who died before closure were not assigned a closure time.

Among the 14 DDAC patients, primary approximation was anatomically not feasible in 8 (57.1%), while intra-abdominal pressure exceeded the 10 mmHg threshold after approximation in the remaining 6 (42.9%). Individual characteristics and outcomes of the 14 patients managed with DDAC are summarized in Table 4. Definitive fascial closure of the abdominal wall was achieved in 9 of 14 patients (64.3%); the remaining 5 patients died after CDH repair but before completion of abdominal wall closure. There were no conversions to an alternative technique of abdominal wall closure. No cases of patch or wound infection, fascial dehiscence, bowel injury, skin necrosis, enterocutaneous fistula or clinically diagnosed abdominal compartment syndrome were observed. Intra-abdominal pressure exceeded 12 mmHg after approximation in two patients, requiring release of the approximation sutures with successful reattempt 24 h later. This transient pressure elevation had no clinically apparent consequences in either patient, including preserved urine output and no signs of end-organ dysfunction. Additionally, no episodes of incisional hernia were observed during the follow-up period in any of the patients. No cases of abdominal compartment syndrome or need for decompression were observed among patients undergoing primary closure.

The rate of acquired thoracic malformations did not differ significantly between DDAC survivors and the primary closure group (2/7 [28.6%] vs. 5/26 [19.2%]; *p* = 0.9). Scoliosis was significantly more frequent in the DDAC group than in primary closure survivors (4/7 [57.1%] vs. 1/26 [3.8%]; *p* = 0.004). Across the entire cohort, scoliosis was identified in 15.2% of patients (5/33).

### 3.3. Factors Associated with Mortality

Bivariable analysis identified the following variables as significantly associated with mortality: DDAC (*p* = 0.001), gestational age (*p* = 0.002), O/E LHR (*p* < 0.001), O/E LHR severity (*p* = 0.021), FETO (*p* = 0.018), HFOV (*p* = 0.003), ECMO (*p* < 0.001), defect type (*p* < 0.001), diaphragmatic closure type (*p* = 0.013) and liver herniation (*p* = 0.005) (Table 5).

**Figure 2:**
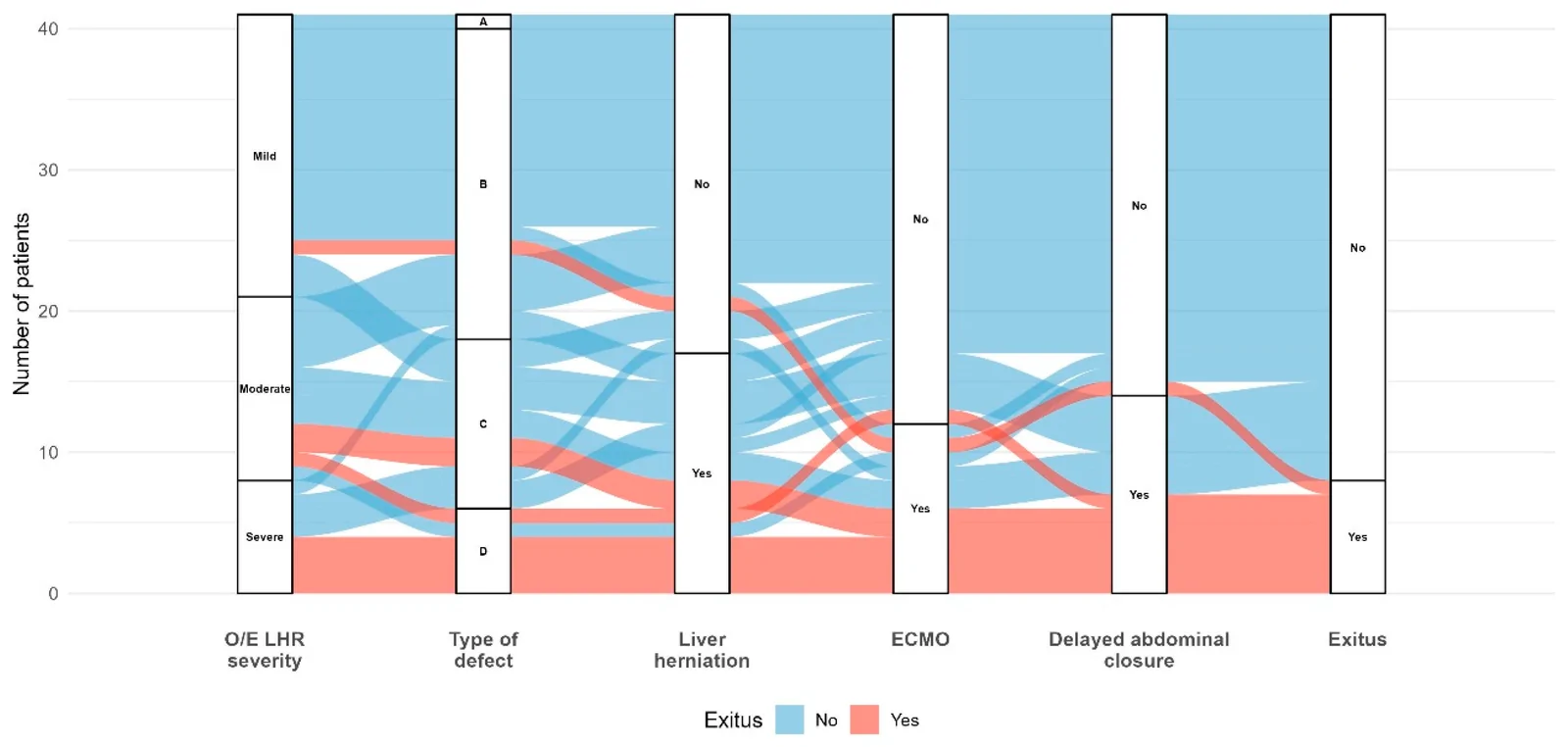
Alluvial plot showing patient flow across key severity variables (O/E LHR severity, ECMO, defect type and liver.

Notably, as illustrated in Figure 2, mortality followed the gradient of disease severity across O/E LHR severity, defect type, liver herniation and ECMO with deceased patients clustering among those with the most severe anatomical and prenatal characteristics. Delayed abdominal closure was consistently associated with this severity profile, suggesting that the observed mortality difference between groups reflects baseline disease burden, although an independent contribution of the surgical technique cannot be excluded from these data.

## 4. Discussion

In this study we present our experience with delayed abdominal closure following CDH repair, introducing a novel technique based on a Gore-Tex^®^ abdominal patch with dynamic traction under intra-abdominal pressure monitoring. To our knowledge, this is the first description of this specific technique in the CDH population. This technique achieved complete abdominal wall closure at a median of 6 days from the surgery. Despite being applied in a significantly more severe patient subset, the mortality difference observed between groups was closely associated with baseline disease severity. However, given the study design, a contribution of the abdominal closure strategy itself cannot be excluded.

The need for delayed abdominal wall closure in CDH repair arises from the inability to achieve primary fascial closure without risking ACS [15]. Treatment of this life-threatening complication involves abdominal decompression through laparotomy, followed by staged fascial closure once hemodynamic stability is achieved [16]. To prevent this complication, continuous intra-abdominal pressure monitoring through a bladder catheter is recommended, with pathological thresholds of 10 mmHg [3,4]. In our cohort, no patient undergoing primary closure subsequently developed ACS, supporting the safety of this threshold as a criterion for selecting patients suitable for primary closure.

Several strategies have been described in the literature to manage patients in whom primary abdominal wall closure is not feasible. Silastic silos or patches sutured to the fascial edges are one of the preferred strategies as they offer the advantage of allowing visual monitoring of bowel perfusion and facilitating gradual visceral reduction as the patient stabilizes. This approach also benefits from the extensive experience gained from visceral reduction in congenital abdominal wall defects [7,8,17,18]. Other documented strategies include vacuum-assisted closure, which aids in peritoneal fluid drainage but requires cautious pressure management to avoid iatrogenically increasing intra-abdominal pressure and skin only closure, which is technically simpler but does not prevent fascial retraction [10].

The key element of our technique is the combination of a Gore-Tex^®^ patch with sequential dynamic traction and continuous bladder pressure monitoring with a safety threshold of 12 mmHg. This approach allows for progressive fascial approximation in a controlled manner, avoiding the consequences of excessive intra-abdominal pressure and the risk of ACS.

The originality of our approach does not reside in dynamic fascial traction per se, which is well established in adult open-abdomen management through mesh-mediated and device-mediated techniques [19,20], nor in the concept of staged reduction with sequential tightening, long familiar from silo management of gastroschisis and omphalocele [21,22]. While Gore-Tex^®^ patches have been previously used for both diaphragmatic and abdominal wall closure in CDH [2,6,18], their use as a dynamic traction device with sequential approximation has not been previously described in this population. The novelty lies in the specific combination of a Gore-Tex^®^ patch with successive traction sutures for delayed abdominal closure in neonates with CDH, a population in which criteria for indicating and closure timing remain poorly standardized.

The time to fascial closure in ECMO patients in our series (median 6.5 [6.00–7.75] days) was shorter than that reported by Laje et al. [10], who described a mean time to fascial closure of 14.5 ± 7 days in patients undergoing CDH repair on ECMO using various coverage strategies including silos, prosthetic mesh, and vacuum-assisted closure devices. This comparison is unadjusted and derives from different cohorts with different case mix and coverage strategies and should therefore be interpreted with caution. Taken together, these findings suggest that early definitive fascial closure prior to hospital discharge is technically feasible in CDH patients requiring delayed abdominal closure. This approach may avoid the morbidity associated with prolonged patch exposure, including patch infection and dehiscence, which have been reported as significant complications in series with longer times to definitive closure, as described by the Mannheim group [6].

Patients requiring DDAC presented significantly more severe baseline characteristics, including lower O/E LHR, higher rates of liver herniation, more advanced defect type and greater need for ECMO and HFOV. This severity profile translated into higher overall mortality in the DDAC group, due to the presence of well-established poor prognostic factors [1]. The association between FETO and both DDAC and mortality likely reflects that fetal therapy is preferentially offered to the most severely affected fetuses. This may also explain the lower gestational age observed in the DDAC group, as FETO is associated with preterm delivery [23]. Mortality was strongly associated with the gradient of disease severity across prenatal and anatomical markers. However, given the retrospective design, small sample size and absence of adjusted analyses, these data do not allow us to exclude an independent contribution of the abdominal closure strategy to mortality, nor can they establish the safety of DDAC relative to other delayed closure techniques, for which no comparator cohort was available in this study.

The overall rate of musculoskeletal deformities in the full cohort was similar to that previously described [1,2,24,25,26]. The higher rate of scoliosis observed among DDAC survivors deserves cautious interpretation. Several biologically plausible mechanisms have been proposed in the literature, including larger diaphragmatic defects requiring patch repair, prolonged mechanical ventilation and liver herniation, all of which were more prevalent among DDAC patients in our cohort and have been individually associated with musculoskeletal deformities in prior series [13,24,25,26]. However, given methodological limitations discussed below, this finding should be regarded as hypothesis generating rather than conclusive.

This study has several limitations. The retrospective and single-center design limits generalizability. Furthermore, the small sample size and low number of events did not allow formal multivariable analysis. The reported time to definitive abdominal wall closure was calculated only among patients who achieved closure, as patients who died before could not be assigned a closure time. Since these patients did not survive long enough to reach this outcome, this form of informative censoring may lead to an underestimation of the time to closure across the full DDAC cohort. Additionally, given that patients requiring DDAC presented with markedly more severe baseline disease, complete separation between groups precluded the use of standard logistic regression. Firth’s penalized likelihood logistic regression was attempted to address this issue; however, the small number of cases resulted in extremely wide confidence intervals, precluding meaningful conclusions and therefore not reported. Propensity-score methods were similarly not feasible given the marked baseline imbalance. Scoliosis and thoracic wall malformations were assessed only when clinically indicated, rather than systematically or at comparable ages. Given that DDAC survivors had more severe disease and closer follow-up, this difference in scoliosis rates may reflect a detection bias rather than a true biological mechanism. Despite these limitations, the consistency of findings across descriptive and bivariable analyses, together with the alluvial plot, supports the feasibility of this technique and a mortality pattern consistent with baseline disease severity in CDH patients in whom primary abdominal wall closure is not feasible. Safety relative to other delayed closure strategies cannot be established from these data.

## 5. Conclusions

DDAC appears to be a technically feasible approach for achieving definitive fascial closure within the first postoperative days in CDH patients unsuitable for primary closure. The higher mortality observed in this group was strongly associated with baseline disease severity, although an independent contribution of the closure technique cannot be excluded and its safety relative to other delayed closure strategies remains to be established.

## Figures and Tables

**Table 1:** Baseline characteristics and postnatal respiratory/ECMO management according to abdominal closure strategy.

	Overall	Primary Abdominal Closure	Delayed Abdominal Closure	*p*-Value
	*n* = 41	*n* = 27	*n* = 14	
**Baseline characteristics**				
Sex, *n* (%)				0.7
- Female	13 (31.7)	8 (29.6)	5 (35.7)	
- Male	28 (68.3)	19 (70.4)	9 (64.3)	
O/E LHR severity, *n* (%)				<0.001
- Mild	20 (48.8)	19 (70.4)	1 (7.1)	
- Moderate	13 (31.7)	6 (22.2)	7 (50.0)	
- Severe	8 (19.5)	2 (7.4)	6 (42.9)	
- Extreme	0 (0)	0 (0)	0 (0)	
O/E LHR	35.00 (28.00–43.90)	42.00 (34.00–46.00)	26.30 (21.40–31.00)	<0.001
FETO, *n* (%)	7 (17.1)	1 (3.7)	6 (42.9)	0.004
Gestational age (weeks)	39.00 (38.00–39.00)	39.00 (38.00–39.00)	37.00 (35.00–39.00)	0.008
Birth weight (g)	3072 (2880–3240)	3100 (2965–3240)	2730 (2290–3300)	0.12
APGAR 1	5.00 (3.00–8.00)	6.00 (3.00–8.00)	4.50 (3.00–6.50)	0.4
APGAR 5	8.00 (6.00–9.00)	8.00 (7.00–9.00)	7.00 (5.00–8.00)	0.050
**Postnatal respiratory and ECMO management**				
HFOV, *n* (%)	21 (51.2)	10 (37.0)	11 (78.6)	0.020
ECMO, *n* (%)	12 (29.3)	3 (11.1)	9 (64.3)	<0.001
***Among patients on ECMO***				
	***n*** ** = 12**	***n*** ** = 3**	***n*** ** = 9**	
Day of ECMO initiation	1.00 (0.00–5.00)	5.00 (3.00–8.00)	0.00 (0.00–1.00)	
ECMO duration (days)	11.00 (9.00–18.00)	11.00 (9.00–11.00)	13.00 (10.00–18.00)	

**Table 2:** Surgical characteristics of patients according to abdominal closure strategy.

	Overall	Primary Abdominal Closure	Delayed Abdominal Closure	*p*-Value
	*n* = 41	*n* = 27	*n* = 14	
Time to surgery (days)	4.00 (3.00–7.00)	3.00 (3.00–6.00)	5.00 (3.00–11.00)	0.2
Hernia laterality, *n* (%)				0.9
- Left	36 (87.8)	24 (88.9)	12 (85.7)	
- Right	5 (12.2)	3 (11.1)	2 (14.3)	
Type of defect, *n* (%)				<0.001
- A	1 (2.4)	1 (3.7)	0 (0)	
- B	22 (53.7)	22 (81.5)	0 (0)	
- C	12 (29.3)	3 (11.1)	9 (64.3)	
- D	6 (14.6)	1 (3.7)	5 (35.7)	
Liver-up, *n* (%)	17 (41.5)	4 (14.8)	13 (92.9)	<0.001
Diaphragmatic closure, *n* (%)				<0.001
- Primary	23 (56.1)	23 (85.2)	0 (0)	
- Patch	18 (43.9)	4 (14.8)	14 (100)	

**Table 3:** Postoperative outcomes of patients according to abdominal closure strategy.

	Overall	Primary Abdominal Closure	Delayed Abdominal Closure	*p*-Value
	*n* = 41	*n* = 27	*n* = 14	
Invasive mechanical ventilation (days)	11.00 (5.00–29.00)	8.00 (5.00–22.00)	33.50 (23.00–63.00)	0.003
Oxygen dependency (days)	24.00 (11.00–57.00)	17.00 (10.00–28.00)	64.50 (41.00–98.50)	<0.001
Time to first enteral feed (days)	6.00 (2.00–9.00)	4.00 (2.00–9.00)	6.00 (5.50–10.00)	0.3
Length of hospital stay (days)	31.00 (17.00–68.00)	30.00 (17.00–42.00)	42.50 (21.00–87.00)	0.3
***Among survivors***				
	***n*** ** = 33**	***n*** ** = 26**	***n*** ** = 7**	
Time to complete enteral feeding (days)	8.00 (5.00–16.00)	7.50 (5.00–10.00)	20.50 (15.50–30.00)	<0.001
Home oxygen requirement at discharge, *n* (%)	7 (20.6)	3 (11.5)	3 (42.8)	0.093

**Table 4:** Individual characteristics and outcomes of patients managed with DDAC.

Patient	Type of Defect	ECMO	Surgery Performed on ECMO	Indication for DDAC	Day of Definitive Fascial Closure	Achievement of Definitive Fascial Closure	Complications	Outcome
DDAC-1	C	Yes	No	Primary approximation not feasible	6	Yes	No	Survived
DDAC-2	C	No	-	IAP > 10 mmHg after approximation	3	Yes	No	Survived
DDAC-3	D	Yes	No	Primary approximation not feasible	-	Deceased before closure	IAP > 12 mmHg. Sutures released	Deceased
DDAC-4	C	Yes	No	Primary approximation not feasible	6	Yes	No	Survived
DDAC-5	C	Yes	No	IAP > 10 mmHg after approximation	7	Yes	No	Deceased
DDAC-6	C	Yes	No	Primary approximation not feasible	-	Deceased before closure	No	Deceased
DDAC-7	C	No	-	IAP > 10 mmHg after approximation	4	Yes	No	Survived
DDAC-8	C	Yes	Yes	IAP > 10 mmHg after approximation	10	Yes	No	Survived
DDAC-9	D	No	-	Primary approximation not feasible	13	Yes	IAP > 12 mmHg. Sutures released	Deceased
DDAC-10	C	No	-	IAP > 10 mmHg after approximation	5	Yes	No	Survived
DDAC-11	C	No	-	IAP > 10 mmHg after approximation	4	Yes	No	Survived
DDAC-12	D	Yes	Yes	Primary approximation not feasible	-	Deceased before closure	No	Deceased
DDAC-13	D	Yes	Yes	Primary approximation not feasible	-	Deceased before closure	No	Deceased
DDAC-14	D	Yes	Yes	Primary approximation not feasible	-	Deceased before closure	No	Deceased

**Table 5:** Baseline and surgical characteristics according to mortality.

	Overall	Survivors	Non-Survivors	*p*-Value
	*n* = 41	*n* = 33	*n* = 8	
DDAC, *n* (%)	14 (34.1)	7 (21.2)	7 (87.5)	0.001
Sex, *n* (%)				0.2
- Female	13 (31.7)	9 (27.3)	4 (50)	
- Male	28 (68.3)	24 (72.7)	4 (50)	
Gestational age (weeks)	39.00 (38.00–39.00)	39.00 (38.00–39.00)	36.00 (34.00–37.50)	0.002
Birth weight (g)	3072 (2880–3240)	3100 (2920–3240)	2705 (2295–3135)	0.13
O/E LHR	35.00 (28.00–43.90)	41.00 (31.00–44.00)	25.50 (20.20–27.50)	<0.001
O/E LHR severity, *n* (%)				0.021
- Mild	20 (48.8)	19 (57.6)	1 (12.5)	
- Moderate	13 (31.7)	10 (30.3)	3 (37.5)	
- Severe	8 (19.5)	4 (12.1)	4 (50.0)	
- Extreme	0 (0)	0 (0)	0 (0)	
FETO, *n* (%)	7 (17.1)	3 (9.1)	4 (50)	0.018
HFOV, *n* (%)	21 (51.2)	13 (39.4)	8 (100)	0.003
ECMO, *n* (%)	12 (29.3)	5 (15.2)	7 (87.5)	<0.001
Hernia laterality, *n* (%)				0.9
Left	36 (87.8)	29 (87.9)	7 (87.5)	
Right	5 (12.2)	4 (12.1)	1 (12.5)	
Type of defect, *n* (%)				<0.001
A	1 (2.4)	1 (3.0)	0 (0)	
B	22 (53.7)	21 (63.6)	1 (12.5)	
C	12 (29.3)	10 (30.3)	2 (25.0)	
D	6 (14.6)	1 (3.0)	5 (62.5)	
Liver-up, *n* (%)	17 (41.5)	10 (30.3)	7 (87.5)	0.005
Diaphragmatic closure, *n* (%)				0.013
Primary	23 (56.1)	22 (66.7)	1 (12.5)	
Patch	18 (43.9)	11 (33.3)	7 (87.5)	

## Data Availability

The raw data supporting the conclusions of this article will be made available by the authors on request.

## Supplementary Materials

The following supporting information can be downloaded at https://www.mdpi.com/article/10.3390/children13091133/s1, Table S1: Patient distribution by year of inclusion and abdominal closure strategy.
