# Supplementary material for: Delayed Dynamic Abdominal Wall Closure Following Congenital Diaphragmatic Hernia Repair Using a Gore-Tex® Patch: A Single-Center Retrospective Experience for a Complex Pediatric Thoracic Disease
Source: Children (Basel). 2026 Aug 24;13(9):1133. doi: 10.3390/children13091133 (PMC13605272; doi:10.3390/children13091133)
Supplement: Supplementary file 1 [file children-13-01133-s001.zip › children-4502822-supplementary.pdf]

Supplementary Materials

Table S1. Patient distribution by year of inclusion and abdominal closure strategy.

	**Overall**	**Primary abdominal closure**	**Delayed abdominal closure**	**p-value**
	**n = 41**	**n = 27**	**n = 14**	
**Year, n (%)**				0.2
2015	3 (7.3)	2 (7.4)	1 (7.1)	
2016	9 (22.0)	6 (22.2)	3 (21.4)	
2017	5 (12.2)	5 (18.5)	0 (0.0)	
2018	3 (7.3)	3 (11.1)	0 (0.0)	
2019	4 (9.8)	1 (3.7)	3 (21.4)	
2020	5 (12.2)	4 (14.8)	1 (7.1)	
2021	4 (9.8)	2 (7.4)	2 (14.3)	
2022	2 (4.9)	0 (0.0)	2 (14.3)	
2023	6 (14.6)	4 (14.8)	2 (14.3)	
